# Roles played by Locally Elected Representatives in Facilitating Multi-Sectoral Action for Health: Evidence from Kerala, India

**DOI:** 10.5334/aogh.4716

**Published:** 2025-07-28

**Authors:** Devaki Nambiar, Jaison Joseph, Hari Sankar D, Gloria Benny

**Affiliations:** 1 Program Director, Healthier Societies, The George Institute for Global Health, New Delhi, India; 2 Conjoint Associate Professor, Faculty of Medicine, University of New South Wales, Sydney, Australia; 3 Conjoint Professor, Prasanna School of Public Health, Manipal Academy of Higher Education, Manipal, India; 4 Research Fellow, The George Institute for Global Health, New Delhi, India; 5 Senior Research Fellow, The George Institute for Global Health, New Delhi, India; 6 Research Assistant, The George Institute for Global Health, New Delhi, India

**Keywords:** Multi-Sectoral Action, primary healthcare, universal health coverage, role of panchayats, inter-departmental coordination for health, leave no one behind, Sustainable Development Goals

## Abstract

*Background:* Multi-Sectoral Action (MSA) for health involves the collaboration of various stakeholder groups within and beyond the health sector and is seen to be critical for the attainment of the Sustainable Development Goals. In Kerala, India, decentralisation reforms have been in place for some time, and we sought to characterise the roles specifically played by locally elected representatives or Local Self-Government (LSG) members, in relation to MSA.

*Methods:* Between July and October 2021, we conducted in-depth interviews with 80 participants from four districts in the southern Indian state of Kerala. Participants were community leaders, healthcare professionals, public health officials and elected members of LSG bodies. After obtaining written informed consent, participants were interviewed about the roles of various stakeholders in implementing primary care reforms with a particular focus on MSA at the grassroots level. The interviews were recorded, translated into English, and thematically analysed by the research team using ATLAS.ti 9.1 software.

*Results:* Participants ranged in age from 35 to 60 years. LSGs played a number of critical MSA roles, including being a gatekeeper for local action; coordinator of departments, sectors and actors (departments of health, revenue, labour, and education departments as well as volunteers); custodian of community, particularly those ‘left behind,’ crisis manager, team builder and advocate even for communities for which they did not have direct responsibility.

*Conclusion:* LSGs were widely seen by implementers as central figures in coordinating MSA for health in Kerala, before and during the COVID-19 pandemic, and in relation to ‘left behind’ groups. The multiplicity of roles played by LSGs suggests the need for flexibility on the one hand as well as the plurality of roles on the other hand, which may be necessary to enable convergence and MSA, particularly at local levels.

## Introduction

Health has pride of place as the third Sustainable Development Goal (SDG), and it is considered a major contributor to sustainable development [1]. The World Health Organization (WHO) has set the triple billion targets to be achieved by 2023, for promoting the health of people by addressing social and other determinants of health through multi-sectoral collaboration [2]. Multi-Sectoral Action (MSA) refers to ‘deliberate collaboration amongst various stakeholder groups (e.g. government, civil society and private sector) and sectors (e.g. health, environment and economy) to jointly achieve a policy outcome’ [3]. In order to achieve the agenda of SDGs by addressing the key social, economic and environmental dimensions of health, a strong focus on multi-sectoral governance and implementation is essential [4, 5]. Effective health governance extends beyond the formal health system, necessitating collaboration with various departments, sectors, the private sector and civil society. This approach fosters the promotion and maintenance of population health through participatory and inclusive strategies [6].

Global evidence suggests mechanisms used for multi-sectoral collaboration for health, which include cabinet/inter-ministerial committees and secretariats [5, 7–11], inter-departmental committees and units [11, 12], parliamentary committees and, recently, the One Health Approach [13, 14]. Examples include the inter-ministerial SDG monitoring and implementation committee of secretaries of 21 ministries in Bangladesh [9]; ministries of finance and/or planning working bilaterally with other ministries in Denmark and Tanzania [15, 16]; thematic clusters of ministries or departments with related portfolios in Pakistan [17], Rwanda, Uganda, Zimbabwe and Zambia [7]; and multi-sectoral set-ups for non-communicable disease (NCD) control in Iran [18] and India [19]. A few countries have taken more comprehensive approaches, such as health-in-all-policies [16, 20], and whole-of-society approaches to intervene on the social determinants involving health civil society, private sector and development partners [15]. The recent extreme weather events due to climate change, and continuing occurrence of zoonotic diseases in South Asia and Sub-Saharan Africa, have further motivated the health system in the region to adopt a One Health Approach based on multi-sectorial collaboration that has achieved some success [21, 22]. Although there is information on structural frameworks, little is known about the processes employed to implement multi-sectoral collaboration. Of the various process-based approaches for multi-sectoral work described by Boston and Gill [23], some evidence is available for information sharing [9, 12, 15, 24], and aligning sectoral activities [9, 15, 24], but less so for resource sharing [9, 15], shared responsibilities or accountability. Similarly, some countries have reported on structures for multi-sectoral collaboration at local levels [12, 9, 25], but information about processes to implement local-level collaboration is scarce.

In India, aligned with the National Health Policy, 2017, the National Multi-sectoral Action Plan for Prevention and Control of Common Non-communicable Diseases was launched to address risk factors associated with NCDs through interventions supported by sectors beyond health [26]. Likewise, there are many other national programs implemented in the country to promote health, nutrition, social well-being and to address the social determinants of health (SDH) by collaborating with departments beyond health. Some examples include the mid-day meal programme by the Ministry of Human Resource Development [27], the Mahatma Gandhi National Rural Employment Guarantee Act under the Ministry of Rural Development [28], the Red Ribbon Express project by the National Aids Control Organization [29] and the School Health Program under India’s National Health Mission [30]. The concept of ‘resource convergence’ has also been of significance in social development policymaking in collaboration with United Nations agencies (UN) and was adopted as a policy initiative by earlier governments [31].

In India, health falls under the jurisdiction of individual states, making state governments primarily responsible for delivering quality healthcare services to the population [32]. In addition, the constitution was amended in the early-1990s to guarantee the decentralise governance to local levels by way of devolving, funds, functions and functionaries. The southern Indian state of Kerala has been ranked as the best state in the country in terms of overall health performance [33], and the Kerala model of healthcare has been commended as an exemplar for other countries to learn from [34]. There exist a series of interconnected and nested governance structures in service of health in the state [35]. These include the State Health and Family Welfare Department, nested within which is the Directorate of Health Services, Directorate of Medical Education, the National Health Mission and National AYUSH Mission with institutional mechanisms extending district level downwards [36]. Kerala is one of the states where the implementation of the Panchayati Raj (Local Government Rule) Act, as per the 73^rd^ Constitutional Amendment for people’s participation in governance, has been successfully undertaken since 1996 [37]. Under this Act, Local Self-Governments (LSGs) play a vital role in governing across sectors at district, block and panchayat levels in Kerala [38]. Along with the responsibilities of 14 line departments, the Panchayati Raj Act of 1994 transferred primary care institutions to the LSGs, wherein the grama panchayats were given Dispensaries, Primary Health Centres and Sub-Centres, Maternity and Child Welfare Centres, immunisation and other preventive measures as well as the Family Welfare and Sanitation programme; Block Panchayat and Municipalities were given charge of Community Health Centre and Taluk Hospitals; and District Panchayat were awarded control over management of District Hospitals, setting up of centres for care of special categories of persons with disabilities and co-ordination of central and state sponsored programmes at district levels [39].

LSGs share responsibility for supplementing health service delivery, in particular by financing projects such as rural infrastructure development, integrated housing schemes, water and sanitation, health, poverty eradication, social welfare and waste management [40]. Apart from the LSGs, public health facilities in the state receive grants-in-aid from the Member of Parliament/Member of the Legislative Assembly of their local areas to improve the quality-of-service delivery through these facilities. The Government of Kerala has devolved managerial and partial disciplinary control over government staff to LSGs, often serving with them in management-related committees. This serves as a kind of feedback loop for local accountability.^1^

Apart from the LSGs, there are other departments in the state that have launched schemes and programs for health, social security and to address the SDH. These include the Kerala Social Security Mission under the Ministry of Social Justice, which provides grants-in-aid schemes to orphanages, persons living with disability, senior citizens and persons with cancer and more [42], and community-based palliative care programmes jointly implemented by the Arograkeralam mission and LSGs [43]. The Aashraya project to rehabilitate destitute families was initiated by Kudumbasree [44]. There is also an array of schemes by the Scheduled Tribes Department and Scheduled Caste Department to offer welfare and support to the population [45–47].

It is less clear, however, how these state and central schemes manifest and iterate at local levels, and indeed how local implementers view and operationalise MSA. We aimed to fill this gap by assembling evidence on the roles played by LSGs in particular in relation to MSA in Kerala State.

## Methods

We conducted in-depth interviews (IDIs) with 56 Health System Actors (HSAs), 17 elected representatives from LSGs, and 7 community leaders. Participants were drawn from primary healthcare facilities across four districts between July and October 2021. The detailed methodology on the selection of districts and facilities is reported elsewhere [48], as this study is the qualitative component of a larger health system research study in Kerala.

Participants for the study were selected and recruited using a purposive sampling approach, as they were the functionaries serving in the multi-stage randomly sampled sites that were selected as part of the parent study. Two primary healthcare facilities (now known as Family Health Centres (FHCs)) per district were randomly selected, and the participants for this study were identified and recruited from these facilities and their corresponding LSGs. We adopted a two-step approach for identifying and selecting participants. Firstly, we created a list of potential HSAs and community leaders eligible for inclusion in the study. This list comprised medical and public health personnel, such as Medical Officers (MOs), Staff Nurses/Nursing Officers, Health Inspectors (HIs), Junior Health Inspectors (JHIs), Public Health Nurses (PHNs), Junior Public Health Nurses (JPHNs), Palliative Care Nurses and Accredited Social Health Activists (ASHAs). For the elected representatives the eligible individuals included Panchayat Presidents, Vice Presidents, Health Standing Committee members and Ward members. Further, we identified community leaders in the sample areas through collaboration with HSAs, LSG members and non-governmental organisations, aiming to gather a broader perspective from the community.

Ethical approval for the study was granted by the Institutional Ethics Committee at the authors’ institute (No. 05/2019). The IDIs were conducted by three researchers trained in qualitative research methods, with each researcher working in a different facility area, and the research team consisted of two male research fellows and one female research assistant, supervised by a senior health system researcher. Administrative approval was obtained from the Department of Health and Family Welfare, Government of Kerala. To secure district level permission, the research team met with the District Medical Officers (DMOs) from four districts, presenting the necessary department permissions, outlining the study’s objectives and sharing findings from the previous round of the study conducted in the same areas. At the facility level, the research team made appointments with MOs, briefed them about the study and took their permission for IDIs with the staff under their institutions. Further, we personally met with each identified HSAs to schedule interviews at their convenience. IDIs were conducted with in-person or via platforms such as Zoom, depending on the participants’ preference. Initial permissions to conduct IDIs were obtained from the respective LSGs. Community leaders were contacted by phone to explain the purpose of the study, and the research team arranged meetings to conduct IDIs based on their availability. All participants received a hard copy of the topic guide and the participant information sheet, available in English and Malayalam, prior to the interviews. Signed informed consents were obtained from each participant, both for their participation and recording the interviews. For those who opted for online interviews, a soft copy of the topic guide and participant information sheet was shared in advance, and their signed consent forms were collected before commencing the interviews.

The IDIs were conducted in the regional language, Malayalam, and lasted between 20 and 60 min. Despite reaching data saturation early for some study topics, all interviews with the pre-identified participants were completed to ensure comprehensive coverage of the perspectives and contexts of the HSAs across various roles and geographical locations within the four districts.

Three participants were unable to take part in the interviews due to their busy schedules, and after multiple unsuccessful attempts to reschedule, they were excluded from the study. All IDIs were recorded, with the recordings and filed notes securely stored in a password-protected database, accessible only to the research team. Transcription and transliteration into English were carried out by a third-party agency approved by the parent research institution, which signed confidentiality agreements before accessing any data.

The translated transcripts were reviewed by a three-member research team to ensure quality and accuracy. Thematic analysis of the transcripts was conducted using ATLAS.ti 9 software by a four-member research team. An inductive approach was employed, with the thematic structure and codebook finalised through multiple discussions amongst the team. Coded manuscripts were then merged with ATLAS.ti 9, with key-coded indexed, and themes were consolidated based on further discussions and the study’s core questions. These questions included the roles of various stakeholders in implementing primary care reforms, coordination of the multi-sectoral coronavirus disease 2019 (COVID-19) response at the grassroots level, and schemes or programs designed for underserved populations. A narrative was subsequently constructed around these themes by the lead author, incorporating inputs, edits and reviews from other authors to ensure clarity and coherence. As we brought together our analysis, we concluded that amongst common theoretical perspectives used to describe MSA, a political economy perspective was the most appropriate as it drew attention to the institutional arrangements, framing and culture of MSA brought together at the LSG level [49]. Further, drawing on our earlier work, this was an example of convergence of decision-making for a particular geographic location, which is also one of the many typologies of convergence or action on health and its determinants [50].

## Results

### Participant characteristics

Data were obtained from a total of 80 participants in the study, of which more than half (60%) were women (see Table 1). Out of the total participants recruited for the study, 70% were HSAs. From this group of participants, we received information on various roles played by LSGs in MSA both in relation to primary care reforms and COVID-19 prevention and control activities in the state.

**Table 1:** Participant characteristics.

CATEGORY	DESIGNATION	FEMALE	MALE	TOTAL
Local Self-Government Representatives	Panchayat President	3	4	7
	Panchayat Vice President	0	1	1
	Health Standing Committee Member	3	5	8
	Ward Member	0	1	1
	Community Leader	1	6	7
Health System Actors	Medical Officer	5	3	8
	Health Inspector	1	5	6
	Public Health Nurse	4		4
	Junior Health Inspector	0	7	7
	Junior Public Health Nurse	11	0	11
	Nursing Officer	3	0	3
	Palliative Nurse	1	0	1
	Community Health Worker	16	0	16
	**Total Participants**	**48**	**32**	**80**

### LSG as gatekeeper

We found that LSG institutions and members (those serving in *Grama Panchayats* or village councils as well as Municipal leaders) were seen as gatekeepers, facilitators and coordinators for addressing the SDH, health-related outcomes and primary healthcare reforms in the state through the support of multiple departments. To begin with, a Community Health Worker noted that primary care reforms in the state would not have been possible with the support of LSGs in Kerala which ‘The Aardram initiative itself is carried out with the help of the Panchayat. No programme is possible without the Panchayat coming to know about it.’ (CHW District 01)

### LSG as coordinator

The primacy of LSG involvement in any programming at the local level is what was envisioned in the Constitutional Amendments of India. This also has a coordinating function, as described by an MO:

….. At a Panchayat level, it is the coordination of multiple departments. Panchayat is a facilitator for all the departments, it brings all departments under a single umbrella. ……. Many of the missions like the Nava Kerala Mission are aimed at ensuring good health and good life. If there are good playgrounds, people will come to play during the evening, and it will decrease NCDs. Similarly, sanitation and waste disposal are all important. It needs to be organised involving all department heads. If we coordinate with all the department heads it will be beneficial for the Panchayat. (MO District 02)

LSG members played a vital role in the functioning of primary healthcare facilities across systems of medicine (even as these are governed and operated quite independently on the government department side), with a number of our participants mentioned. Critically, this involved engagements with providers at various levels, matching jurisdictions of health functionaries. As stated by a JPHN:

We are in touch with the ward members. They sort out these issues. The Medical Officers and other such officers are in constant contact with the Panchayat. The JPHNs and JHIs are more in contact with the ward members. (JPHN District 03)

### LSG as custodian of community, particularly those ‘left behind’

The legacy and genesis of LSG governance in the state was also underscored as ensuring that community priorities were developed. According to one MO:

The Local Self Governments cannot refrain from doing any activities because these activities are required by the people often. For example, medicines for Non-Communicable Diseases, palliative care - these cannot be avoided. …We have almost 18 ongoing health projects. The main reason is the involvement of the Panchayat. When good projects are introduced, we recognise them and discuss with our staff about possible involvement. This time, we have covered all areas, including migrants and elderly people. We have special programs for migrants, adolescents, the elderly, and TB patients. This is the success of peoples planning and decentralisation. (MO District 02)

This was especially the case for groups facing disadvantage, which required wrangling schemes and programming across departments at the local level and systems, at times in short order. This was observed by a JHI, who noted with respect to tribal areas that

the District Tribal Department used to organise mobile medical camps there. In addition to that, we also conduct[ed] camps including NCD clinics. Some COVID cases were reported there so we made everyone undergo RT-PCR tests. We also have a system to deliver food and other things with the help of the ward member if they have any such difficulty. (JHI District 02).

Home delivery of medicines took place not just for allopathic/biomedical drugs but also for other systems of medicine. This was of particular use and relevance to palliative care patients, for whom home-based care had already been the norm under LSG-funded programs.

### LSG as crisis manager

The precedent set by LSGs in coordinating departments as part of health programming was seen to set a precedent of working together during the COVID-19 pandemic. As indicated by an MO, ‘Coordination in Aardram has been done very well during these COVID times - revenue, police, panchayat; everyone has been working with the single goal of stopping this pandemic.’ (MO District 04). In many cases, routine meetings held by LSGs allowed groups to work together as a team and allocate roles to manage pandemic responsibilities. With specific reference to the imperative of reducing test positivity rates, a ward member indicated that

After the positive cases increased, every week, on Fridays, the Panchayat has began to conduct a review session in connection with reducing the TPR rate. We get in touch with the police officers, health inspector, medical officer, and a leadership team from the Panchayat. ...Panchayat give instructions and assign Rapid Response Team [RRT] members - 10–15 members for each ward - under a JHI and ASHA workers, and there were also voluntary workers at the ward level. (WM District 03)

### LSG as team builder (particularly for those facing disadvantage)

Attention was paid to the mechanics of teamwork in a pandemic context with unprecedented innovations and interventions also being brought together by LSG leaders. The simple provision of a school vehicle which was under the jurisdiction of the education department but that an LSG member could marshal for other purposes, allowed MSA teams to work together. This unique innovation was noted by a JHI from another district:

We were able to provide services more than what we had planned at the field level. At the beginning itself, a school vehicle was provided to us. This is something no panchayat has done. They gave us vehicles, panchayat staff for support, police staff, revenue staff, Medical Officer, public health officials, everybody together. It was a big vehicle; everybody was able to sit while maintaining social distancing. After going like that, we were able to provide them with medicines and other facilities. (JHI District 01)

We also found a number of joint initiatives implemented by the LSGs, which brought together the Kudumbasree^2^ scheme (under the Local Self Government Department) and health staff in the Primary Health Center (PHC) focussing on the elderly population, migrants, destitute and palliative care patients by way of extending the scope of schemes. A Community Health Worker described an example of this:

Through the Ashraya project, the Panchayat supported people who are isolated. It ensured that nobody goes hungry. The Panchayat came to aid all such people, included them within the Ashraya project and provided them with essential ration every month. Nobody was ignored or excluded. (CHW District 03)

The support of Kudumbashree workers was linked up to other teams and initiatives and appreciated by LSG members:

We had the support of Kudumbashree workers. …The workers never failed in providing food items to the houses where someone was COVID positive. This was done using Janakeeya (community kitchens introduced during COVID-19 by the government to offer subsidized meals) hotels. Services were delivered on time without any delays. Kudumbashree workers, RRT members, HPT (Health Promoter Teams) all worked well. (Panchayat President District 04)

### LSG as advocate

We found that LSGs have the capability of identifying local needs even in adjacent areas and seeing their addressal, which a JHI noted:

Even though the XX Panchayat and the YY Panchayat are situated nearby, both have different needs. One Panchayat might need more of a particular facility and the other panchayat might not require the same facility quite as much. So, with the coming of Local Self Government Departments, it became much easier to address these disparities and come up with solutions much quicker. (JHI District 03)

These various characteristics of LSGs were observed both prior to and in the pandemic context and were seen as major enablers of MSA.

### Challenges associated with the multiplicity of roles LSGs play

While acknowledging the supportive role offered by the LSGs, participants expressed their concerns over the overreach of LSG members into operational aspects of the health system. An MO emphasised the need for hospitals being politically neutral zones and ascertained that the party politics that come along with LSG involvement should not interfere the day-to-day functioning of health facilities. An MO noted that

They have a supporting role. In my opinion, it should be limited to that. In many places it has grown into an encroachment. Especially due to COVID and the vaccination work. We do not have any politics here. When someone is working in a hospital, irrespective of their political positions, they are healthcare professionals. So, to avoid that, what I expect is to stay partially detached from the Panchayat. If we need any help we will approach them. We will attend their meetings. If needed, the Medical Officer itself will attend. Otherwise, the clerk or HI will attend. Whichever way, we will be represented. It should be limited. I do not support a Panchayat rule inside a hospital. (MO District 02)

HSAs reflected the increased involvement of LSGs in community-level outreach activities post COVID-19 vaccination campaigns; this was in some contexts seen as positive and in some instances as problematic. Community health workers highlighted the issues of equity and transparency in COVID-19 vaccination. Recalling the incidents of LSG members and their affiliates accusing them of favouritism (towards populations prioritised for COVID vaccination) or in turn demanding favouritism bypassing the official protocols (like online appointment booking) was reported, and was seen to strain relationships and politicise health services:

Vaccination is given as per online booking, and they (LSG members) would ask Slots for people who have not registered to get vaccinated on that day. These would be a small number of people, and whether it may be political or otherwise, there are people that give us trouble regarding this. They are not allowing us to give vaccinations. Their main complaint is that we are purposely setting them aside. We would never do that. We were given instructions from the Medical Officer as well the HI that we should prioritise people who have diseases as well as elderly people. (CHW District 02)

An LSG president also reflected that the design of decentralisation can sometimes lead to fragmentation of administrative structures and myopic approaches to development. Elected ward members at times were reportedly more focussed on developing their own ward rather than their parent Panchayat area, which could lead to conflicts in deciding on utilising allocated funds. They said: ‘*There have been some administrative issues from the part of public representatives too. Majority of these people will not become Panchayat members. They remain as ward members.*’ (LSG District 01). Here, the participant was suggesting that such a narrow view also limited the political dividend and electoral potential of the local leader. Such challenges were therefore seen from the perspective of both health system and LSG actors.

## Discussion

Our study sought to characterise implementer perspectives on MSA mechanisms in relation to LSGs in Kerala. LSGs had critical important features and roles seen as critical for MSA, as envisaged and specifically instantiated in the state’s healthcare reform programme called the Aardram mission [51]. Implementers were largely aligned with the value and contributions of LSG actors in converging health-related services, acting purposefully during a pandemic and reaching those left behind.

Our analysis of implementer perspectives reveals a common mental frame amongst health department functionaries, and LSG members themselves as well as community leaders feel that LSG plays a vital role in the functioning of the primary healthcare facilities irrespective of the system of medicine. This kind of ‘co-framing’ is seen to be essential to align politics and enable multi-sectoral coordination, for instance in obesity prevention [52].

The larger context appears to matter as well, for such roles of LSGs to emerge. A study by Ghosh et al. (2019) reports that AYUSH systems (Ayurveda, Yoga and Naturopathy, Unani, Sidha and Homoeopathy) in Kerala have effectively utilised the decentralisation of power through the LSGs, which have increased access to the public health centres facilitated by the department of AYUSH along with LSGs [53]. In India, engaging LSGs in health has been recognised as the only mechanism to achieve large-scale community participation and to reach the last mile [54], which we found to be functional in Kerala through our study. Along with it, institutional and governance mechanisms like Hospital Management Committee (HMCs) are constituted under each PHC in the state to keep constant vigil on the working of institution, to ensure the steady development of the institution and to take up certain responsibilities for the improved functioning and enhancement of the PHC [55].

Emphasis was placed, in our study, on Aardram healthcare reforms, which is one of the four convergence missions under the ‘Nava Kerala Karma Paripadi’ (New Kerala Action Plan). The other missions are related to green agriculture and ensuring biodiversity (Haritha Keralam (Green Kerala)), Livelihood Inclusion and Financial Empowerment, and education reform [56]. In addition to these missions, the state is advancing the initiative on localising SDGs through LSGs, with targets at the grama panchayat level [57]. A key area of future research will be how these various convergence missions inter-relate and are managed by the same set of actors on the ground. Studies in India mostly speak of MSA in single issue areas (like One Health, NCDs, nutrition or drowning), where convergence has been pursued [58–61]. However, given the type of role envisioned for LSGs in Kerala, they are responsible for various convergence platforms—which may create some challenges for prioritisation.

Pandemics also introduced new opportunity structures for MSA, as we found in our study. Our study found that multiple departments were coordinated by LSGs in managing COVID-19 Pandemic. Kerala’s LSGs took on a range of additional roles, including running awareness programs, such as ‘Break the Chain’ initiative, conducting additional sanitation and cleanliness drives, regular outreach to home isolated/quarantined persons, activating volunteer groups and more, which involved coordinating primary healthcare facilities, police, other departments (civil supplies, education, revenue, women and child, agricultural, SC/ST, etc.), Rapid Response Teams (RRTs) and various other civil society organisations to identify and help groups facing unique vulnerabilities vis-à-vis COVID-19 [62]. There is evidence to suggest that the MSA approach in this context provided much-needed nutrition, healthcare and outreach [63, 64]. In addition, multi-disciplinary monitoring committees formed at the LSG level ensured awareness generation, roll out of preventive measures, essential service delivery, disaster mitigation, convergence and grievance redressal [65]. While looking at the COVID-19 vaccination drive, LSGs linked awareness generation to vaccination drives, such that there was no reported COVID-19 vaccine wastage in the state [66]. How such modalities function outside a pandemic context warrants further study. Further, the way impacts of these initiatives were tracked, monitored and publicly available is not the case with routine programming cycles. Making such information publicly available would yield much-needed insights on impacts of MSA outside a pandemic context.

There has been a very high degree of MSA and coordination to reaching the ‘vulnerable’ in Kerala, which has also been recognised in global fora (for example, in 2019, the state received commendation from the UN for ensuring free treatment and healthcare services for its population through MSA in prevention and control of NCDs, lung diseases, cancer care, control of paralysis and mental health focussing on achieving the SDGs) [67]. Our study participants reflected on activities through inter-departmental convergence as the major mechanism to reach out to the vulnerable population. This particularly manifests in the case of public health insurance in the state, where LSG leaders and Kudumbashree mission workers under the Local Self Government Department played a major role in spreading information about and improving enrolment rates under this scheme implemented by the Department of Labour [68].

Similar mechanisms have been observed for other state-sponsored schemes rolled out in Kerala through Kerala Social Security Mission targeting various vulnerable sections of the society, wherein the LSGs have played a vital role in disseminating information and the Department of Health and Directorate of Medical Education ensures services to the eligible beneficiaries [69, 70]. There is less attention to how these populations have identified priorities, the degree of their effective coverage, and their own experiences and feedback regarding these schemes. This is a critical area of further study. While there is value added by national-level studies on design and goals, greater attention to mechanisms and outcomes of MSA at the block and district level—centre staging the perspective of citizens and beneficiaries—would be important to carry out in Kerala and, for comparison, other parts of India.

The plurality of roles played by LSGs, including in large-scale reform as well as in a pandemic context, has not been without challenges, and HSAs raised concerns over the influence and interference of LSGs and commented that some of these may be politically motivated. Our findings resonate with Sreenidhi et al., who reported that LSGs tend to use health sector activities to attain political mileage and prioritise service delivery amongst favourable groups politically close to them over others [71]. The issue of ‘elite capture’ in participatory decentralised planning of development in health and other sectors continues to exist in Kerala as reported by existing literature [72]. This is a constant negotiation that both shapes and at times hampers the equity of MSA in the state.

## Limitations

In the current study, the perspective of health workers in the modern medicine health sector was only taken. We did not focus on understanding the perspective actors on MSA from other systems of medicines, such as Ayurveda and Homoeopathy that are also governed by LSGs. Our study is exclusively on the MSAs at the grama panchayat level, and our sample did not cover perspective on MSAs at block, district panchayat and other urban local bodies. Finally, and most critically, this analysis does not include perspectives of communities served by LSGs or in particular populations facing disadvantage. Their perspectives would be critical to understand the nature and impact of MSA in the state; this perspective is being compiled in another manuscript based on our parent study.

## Conclusion

Our study found that LSGs are widely seen by implementers as central figures in coordinating MSA for health, both outside and in the context of COVID, and in relation to ‘left behind’ groups. The multiplicity of roles played by LSGs suggests the need for flexibility on the one hand and the plurality of roles on the other hand, which may be necessary to enable convergence and MSA, particularly at local levels. Whether all these roles are necessary, the politicisation of service delivery is a reported challenge. Further, which roles offer added benefit and mitigate politicisation is a subject of further study, which may require cross-case comparison with such leadership structures in other parts of India and the world. Further research should report on the perspectives of intended beneficiaries of MSA programming.

## Figures and Tables

**Table:** 

ASHA	Accredited Social Health Activist
CFLTC	COVID First Line Treatment Centres
CHW	Community Health Worker
DCC	Domiciliary COVID Care Centre
DMO	District Medical Officer
FHC	Family Health Centre
HI	Health Inspector
HMC	Hospital Management Committee
HPT	Health Promotion Team
HSA	Health System Actor
IDI	In-Depth Interviews
JHI	Junior Health Inspector
JPHN	Junior Public Health Nurse
LSG	Local Self-Government
MO	Medical Officer
MSA	Multi-Sectoral Action
NCD	Non-communicable Disease
PHC	Primary Health Centre
PHN	Public Health Nurse
RRT	Rapid Response Team
SDG	Sustainable Development Goals
SDH	Social Determinants of Health
WHO	World Health Organization

## Data Availability

All datasets used to support the conclusions of this paper are available from the corresponding author on request.

## Footnotes

1 A typical example of this is Kerala’s Hospital Management Committee (HMC) which exists for each public facility across modern medicine, Ayurvedic and Homoeopathic systems [41]. The HMC is chaired by the President of the concerned LSG and includes other medical staff from the facility as well as other members of the community [41]. Through the mechanisms of the HMC, LSGs carry out overall management of public health institutions, such as allocations for maintenance of existing infrastructure including building and equipment, building up new infrastructure, purchase of certain medicines, equipment, as well as grievance redressal for patients and the public [41].

2 Kudumbashree is a poverty eradication and women’s empowerment programme implemented by the State Poverty Eradication Mission (SPEM) of the Government of Kerala. For more information, see https://www.kudumbashree.org/#.
